# Density Functional Theory Study of B, N, and Si Doped Penta-Graphene as the Potential Gas Sensors for NH_3_ Detection

**DOI:** 10.3390/membranes12010077

**Published:** 2022-01-08

**Authors:** Guangjun Chen, Lei Gan, Huihui Xiong, Haihui Zhang

**Affiliations:** 1 Faculty of Materials, Metallurgy and Chemistry, Jiangxi University of Science and Technology, Ganzhou 34100, China; Chengj571218@hotmail.com (G.C.); ganlei2005@gmail.com (L.G.); 2 Jiangxi Advanced Copper Industry Research Institute, Yingtan 335000, China

**Keywords:** doped penta-graphene, adsorption, first-principles calculation, gas sensor

## Abstract

Designing a high-performance gas sensor to efficiently detect the hazardous NH_3_ molecule is beneficial to air monitoring and pollution control. In this work, the first-principles calculations were employed to investigate the adsorption structures, electronic characteristics, and gas sensing properties of the pristine and B-, N-, P-, Al-, and Si-doped penta-graphene (PG) toward the NH_3_, H_2_S, and SO_2_ molecules. The results indicate that the pristine PG is insensitive to those toxic gases due to the weak adsorption strength and long adsorption distance. Nevertheless, the doping of B, N, Al, and Si (B and Al) results in the transition of NH_3_ (H_2_S and SO_2_) adsorption from physisorption to chemisorption, which is primarily ascribed to the large charge transfer and strong orbital hybridizations between gas molecules and doping atoms. In addition, NH_3_ adsorption leads to the remarkable variation of electrical conductivity for the B-, N-, and Si-doped PG, and the adsorption strength of NH_3_ on the B-, N-, and Si-doped PG is larger than that of H_2_S and SO_2_. Moreover, the chemically adsorbed NH_3_ molecule on the N-, B-, and Si-doped PG can be effectively desorbed by injecting electrons into the systems. Those results shed light on the potential application of PG-based nanosheets as reusable gas sensors for NH_3_ detection.

## 1. Introduction

In recent years, the excessive emission of poisonous ammonia (NH_3_) has brought a serious threat to the ecological environment and human health [1]. Long-term exposure to NH_3_ can cause bronchitis, lung swelling, and even death [2]. In this regard, the highly efficient detection of trace NH_3_ gas is regarded as an effective method to restrain its negative effects. Nowadays, many researchers have focused on the fields of seeking the high-performance sensing materials for NH_3_ detection [3,4,5]. Among them, the two-dimensional (2D) nanomaterials have great potential application in gas senors due to the good stability, large surface-to-volume ratio, and massive active sites [6,7,8,9]. For instance, C-vacancy defected Ti_2_CO_2_ monolayer was predicted by the first-principles method to be a reusable gas sensor for NH_3_ detection [10]. In addition, NH_3_ adsorption based on borophene [11], C_2_N monolayer [12], phosphorus carbide monolayer [13], and Ni-doped InN monolayer [14] has been theoretically studied to evaluate the possibility of those materials as an NH_3_ sensor. However, some intrinsic shortcomings, including the insufficient sensitivity, excessively large adsorption strength, and long recovery time, restrict their industrial utilization. Thus, it is necessary to explore the high-performance sensor material for NH_3_ detection.

Penta-graphene (PG), a new 2D allotrope of graphene, was recently proposed and predicted to have excellent dynamical and mechanical stability up to 1000K [15]. Moreover, many theoretical investigations showed that PG has a great potential application in gas capture and sensing [16,17,18], hydrogen storage [19], and lithium-ion batteries [20] due to its good mechanical, electrical, and optical properties [21,22]. Cheng et al. [23] investigated the electronic and transport properties of NH_3_, NO, and NO_2_ adsorbed on PG monolayers by using the first-principles and non-equilibrium Green’s function methods, and revealed that PG was highly sensitive and selective to NO and NO_2_ molecules, while it showed poor NH_3_ sensing performance because of the weak physisorption. As known, the heteroatom dopant method has been proven to significantly improve the interactions between gas molecules and substrate [24,25]. For example, the adsorption strength, charge transfer, and sensing properties of CO and CO_2_ on Fe-doped PG were higher than those on the pristine PG [26]. By using the density functional theory (DFT) calculations, Chen et al. [17] found that the doping of B and N into PG could obviously enhance the adsorption strength of SF_6_ decomposed products and demonstrated the B-doped PG could be a good sensing material for H_2_S molecules. In addition, the N-doped PG exhibited high CO_2_ selectivity from the mixtures of H_2_, N_2_, and CH_4_ in an external electric field [16]. In addition, the Pt-doped PG was reported to have good detection ability of H_2_ molecules [27]. Nevertheless, the investigations into the sensing properties of PG with different dopants toward the poisonous NH_3_ are still lacking.

Generally, the gaseous pollutants of NH_3_, H_2_S, and SO_2_ are simultaneously released from the combustion of fuel and industrial production. Therefore, the first-principles calculations based DFT method were performed to study the adsorption behaviors of these toxic gases on the pristine and B-, N-, P-, Al-, and Si-doped PG monolayers, and the corresponding sensing characteristics were discussed in this work. Meanwhile, the adsorption structure and electronic properties (e.g., energy band, density of states, difference charge density, and charge transfer) were also systematically analyzed. Our results indicate that the B-, N-, and Si-doped PG nanosheets could be promising NH_3_ sensing materials with high selectivity and sensitivity, which provide the fundamental basis for the development of novel PG-based gas sensor.

## 2. Calculation Method and Details

All the gas sensing simulations on pristine penta-graphene (PG), doped-PG, and charged PG were performed using the Dmol^3^ module [28] of Materials studio software (Accelrys, California, USA). The generalized gradient approximation in the form of the Perdew–Burke–Ernzerhof [29] and the double numerical plus polarization (DNP) basis set [30] were chosen for all the spin calculations. The Grimme approach [31] and DFT semi-core pseudopotential (DSSP) method [32] were used to describe the van der Waals interactions and core treatment, respectively. The 3 × 3 ×1 supercell of PG (54 atoms) with 20 Å along the z direction was adopted during the simulations, and the Monkhorst−Pack meshes of 4 × 4 ×1 (10 × 10 ×1) were employed to conduct the geometry optimizations (electronic properties calculations), and the Hirshfeld charge was calculated to analyze the charge transfer between gas molecules and substrates. In addition, the global orbital cutoff radius was set as 4.6 Å, and the convergence tolerances were 1.0 × 10^−5^ Ha, 0.002 Ha/Å, and 0.005 Å for total energy, maximum force, and maximum displacement, respectively. In doped PG systems, the formation energy (E_for_) of B, N, P, Al, or Si atoms could be determined by the following equation [17]:

(1) $$E_{\mathrm{for}}{=E}_{\mathrm{doped}\text{-}\mathrm{PG}\;}{-\;E}_{\mathrm{PG}}{+E}_{C}\;{-\;E}_{\mathrm{doped}\text{-}\mathrm{atom}}$$

where $E_{\mathrm{PG}}$ and $E_{\mathrm{doped}\text{-}\mathrm{PG}}$ are the total energies of pristine and doped PG, and $E_{C}$ and $E_{\mathrm{doped}\text{-}\mathrm{atom}}$ are the chemical potential of C and doped atoms, respectively. The chemical potentials of C (B, P, Al, and, Si) were obtained from the average energy for every C (B, P, Al, and Si) atom in bulk diamond (B, P, Al, and Si), while the chemical potential of the N atom was calculated by the N_2_ molecule.

The adsorption characteristics of NH_3_, H_2_S, and SO_2_ over pristine and doped PG were studied in this work, and the adsorption energy (E_a__ds_) could be obtained by [33,34]

(2) $${\;E}_{\mathrm{ads}}{=E}_{\mathrm{gas}+\mathrm{substrate}\;}{-\;E}_{\mathrm{substrate}}\;{-\;E}_{\mathrm{gas}}$$

where $E_{\mathrm{gas}+\mathrm{substrate}}$ and $E_{\mathrm{substrate}}$ are the total energies of pristine PG (doped PG) with and without gas adsorptions, and $E_{\mathrm{gas}}$ is the energy of a single gas molecule. As known, a more negative E_ads_ means a stronger gas–substrate interaction.

## 3. Results and Discussion

### 3.1. Electronic Properties and Stability of Doped PG 

**Figure 1:**
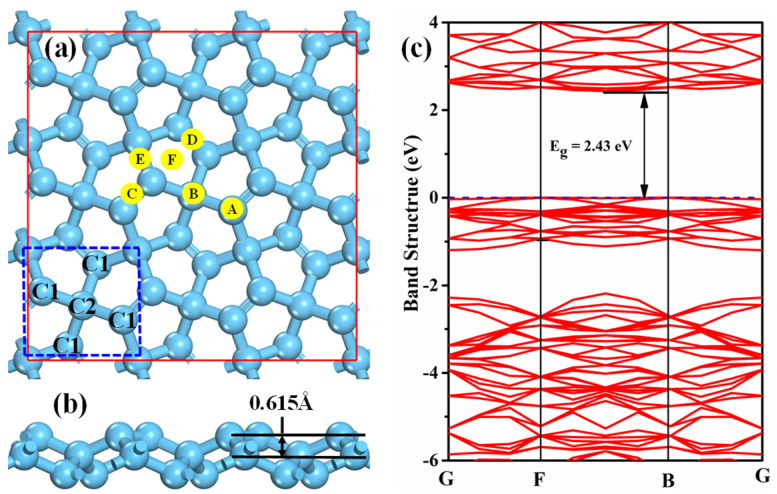
Top view (**a**), side view (**b**), and band structure (**c**) of pristine penta-graphene after full relaxation. The blue dashed square is the primitive cell, and the symbols of A∼F represent the possible adsorption sites.

The optimized lattice constant of PG with P-421M symmetry is 3.63 Å, and the calculated band gap is 2.43 eV (Figure 1c), both of which match well with previous reported results [35]. There are two types of carbon atoms, i.e., the sp^2^ hybridized carbon atom (C1 site) and sp^3^ hybridized carbon atom (C2 site), as shown in Figure 1a. Thus, the C1 and C2 sites substituted by B, N, P, Al, or Si atoms are both taken into account, and the obtained formation energy of different doped PG is listed Table 1. The difference between our results and other studies may be ascribed to the different supercell size and dopant concentration. One can see that N and P substitutions at the C1 site are the exothermic process, while the other doping cases are the endothermic process. Additionally, all the dopant atoms prefer to substitute the C1 atom due to the smaller formation energy, which is consistent with previous calculated results [17,21]. Therefore, the cases of B, N, P, Al, and Si substituting the C1 site are discussed in the following sections.

### 3.2. Adsorption of NH_3_, H_2_S and SO_2_ on Pristine PG

**Figure 2:**
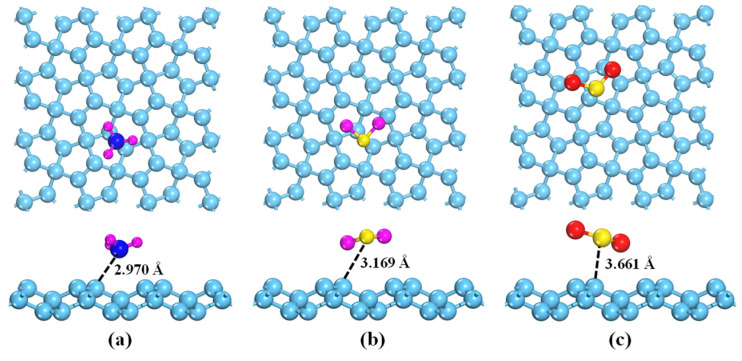
The lowest-energy structures of gas molecules adsorbed on pristine PG: (**a**) NH_3_, (**b**) H_2_S, and (**c**) SO_2_. The light blue, purple, dark blue, yellow, and red balls are C, H, N, S, and O atoms, respectively.

In order to evaluate the detection ability of PG, the adsorption behaviors of NH_3_, H_2_S, and SO_2_ on the pristine PG were investigated in this section. Considering the structure symmetry of PG, six possible adsorption sites, including two top sites (labeled as A and B), three bridge sites (labeled as C, D, and E), and one hollow site (labeled as F) were taken into account, as displayed in Figure 1a. For convenience, one gas molecule adsorption on the PG is defined as “gas@PG”, for instance, NH_3_@PG means the NH_3_ adsorption on PG. The calculated adsorption energy, adsorption distance, charge transfer, and band gap of three toxic gases adsorbed on PG are presented in Table 2. It is found that the adsorption energies of the NH_3_, H_2_S, and SO_2_ molecules are −0.298 eV∼−0.382 eV, and the adsorption distances are in the range of 2.661 Å to 3.169 Å (Figure 2). This indicates that all the toxic gases belong to the representative physisorption, which can be also demonstrated by the small Hirshfeld charge transferred from PG to gases. Moreover, compared with the pristine PG, the band gap of PG after gas molecule adsorptions shows little change. Therefore, the pristine PG exhibits poor detection ability toward the NH_3_, H_2_S, and SO_2_ molecules due to weak interactions and negligible resistance variation.

**Figure 3:**
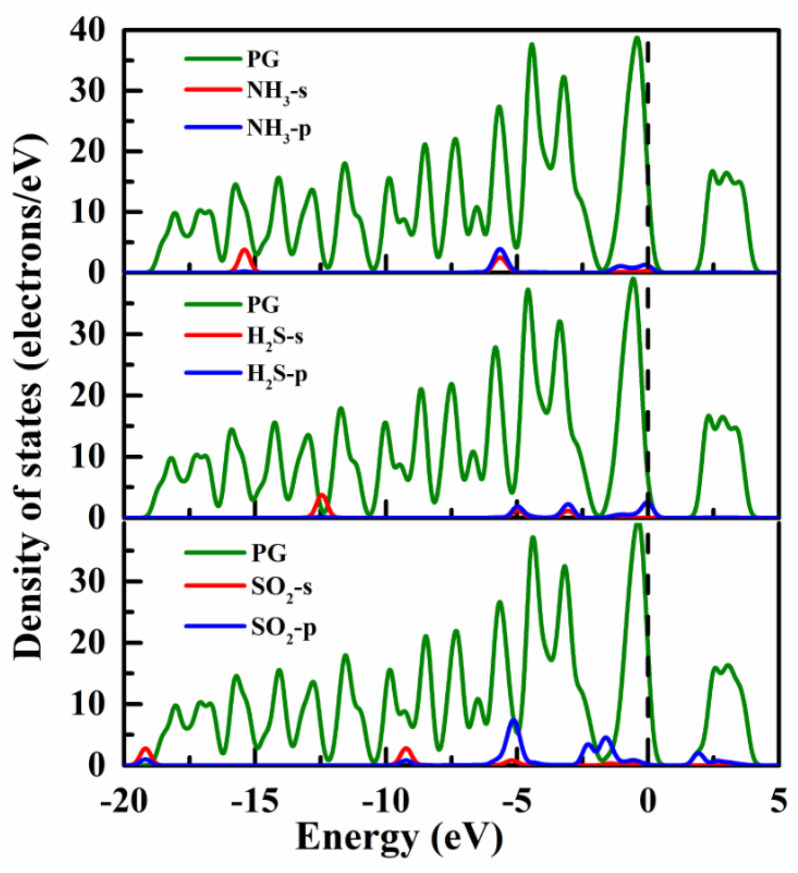
Density of states of NH_3_, H_2_S, and SO_2_ adsorbed on the pristine PG. The dashed line is the Fermi level.

**Figure 4:**
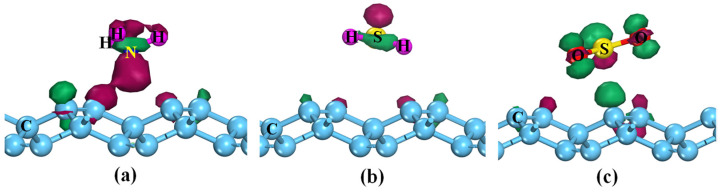
Charge density difference of different adsorption systems (**a**) NH_3_@PG, (**b**) H_2_S@PG, and (**c**) SO_2_@PG. The green (red) region is electron accumulation (depletion), and the isosurface is ±0.01 e/Å^−3^.

To further comprehend the micro-interaction mechanism between gas molecules and PG, the density of states (DOSs) and charge density difference (CDD) of various adsorption systems were calculated, as shown in Figure 3 and Figure 4. For the NH_3_@PG, H_2_S@PG, and SO_2_@PG, the gas–substrate interactions are mainly contributed by the weak hybridizations between PG and p orbitals of gas molecules. By contrast, NH_3_-s, H_2_S-s, and SO_2_-s orbitals show the weaker interactions with the penta-graphene due to the small peak value of DOSs (see Figure 3). For the NH_3_@PG and H_2_S@PG, the partial electrons of NH_3_ or H_2_S are transferred to PG (Figure 4a,b), indicating the NH_3_ and H_2_S molecules act as electron donors, which agrees well with the results of the Hirshfeld charge (Table 1). In addition, compared with the case of H_2_S, NH_3_ loses more electrons and thus has the lower adsorption energy. For the SO_2_@PG, a part of the charge of PG is transferred to the SO_2_ molecule, as shown in Fig. 4c, which suggests the SO_2_ molecule acts as the electron acceptor. However, the small transfer charge between gas molecules and PG still results in weak interactions, and thus the pristine PG shows poor detection ability toward these toxic gases.

### 3.3. Adsorption of NH_3_, H_2_S, and SO_2_ on Doped PG

**Figure 5:**
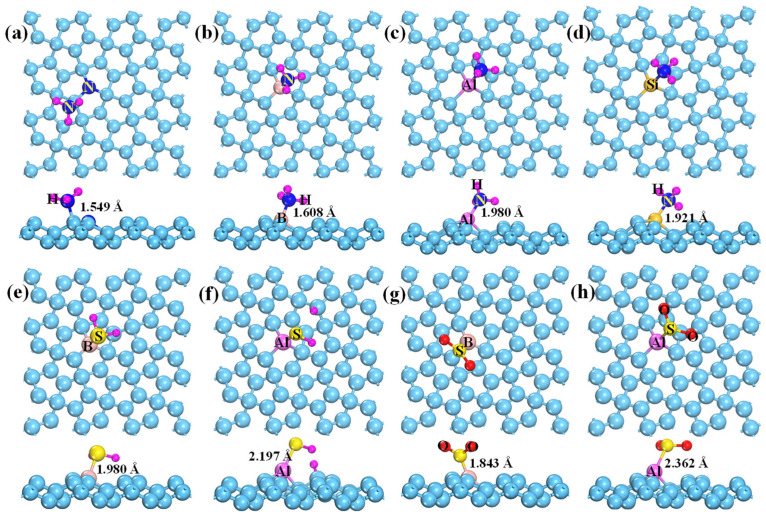
Fully-relaxed structures of different adsorption systems. NH_3_ adsorbed on N-PG (**a**), B-PG (**b**), Al-PG (**c**), and Si-PG (**d**); H_2_S adsorbed on B-PG (**e**) and Al-PG (**f**); SO_2_ adsorbed on B-PG (**g**) and Al-PG (**h**).

The adsorption behaviors of NH_3_, H_2_S, and SO_2_ on M-doped penta-graphene (M-PG, M=B, N, Al, and Si) were also investigated, and the obtained calculation results and corresponding lowest energy configurations are displayed in Table 2 and Figure 5. Compared with the NH_3_ on pristine PG, doping B, N, Al, or Si atoms into PG nanosheets can obviously enhance the NH_3_ adsorption strength with the E_ads_ of −1.06 eV∼−2.46 eV, resulting in the transition of NH_3_ adsorption from physisorption to chemisorption. In addition, the adsorption distances are significantly shortened (Figure 5a–d), and the charge number transferred from NH_3_ to the doped PG is remarkably increased in comparison with the case of NH_3_ adsorption on the pristine PG. By contrast, only the doping of Al and B atoms can strengthen the adsorption of PG toward H_2_S and SO_2_, the rest of the dopants show a slight change (see Table 2). In particular, the H_2_S is dissociation-chemisorbed on the Al-PG surface with one H bonding to the nearest neighbor C atom, as shown in Figure 5f. Therefore, the doping of B, N, P, Al, and Si atoms may be able to improve the detection ability of the NH_3_, H_2_S, and SO_2_ molecules. 

**Figure 6:**
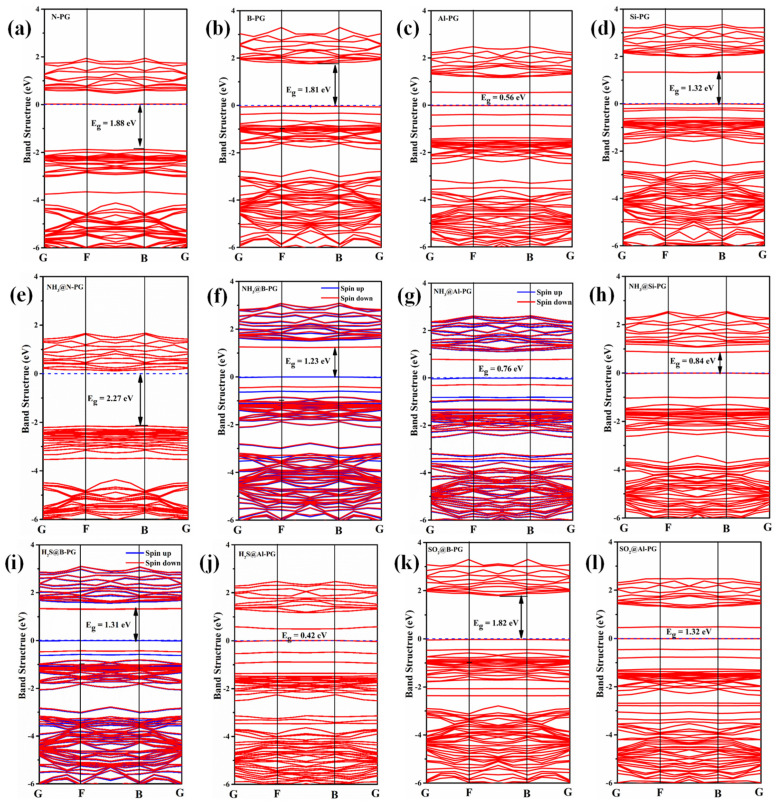
Band structures of the N-, B-, Al-, and Si-doped PG without gas adsorptions (**a**–**d**) and with NH_3_, H_2_S, and SO_2_ adsorptions (**e**–**l**). The dashed lines are the Fermi level.

The variation of electric conductivity (σ) could be utilized to evaluate the sensitivity of a material before and after molecular adsorption, which is defined as σ ∝ exp(−E_g_/2K_B_T), where E_g_, K_B_, and T are the band gap, Boltzmann constant, and temperature, respectively. As mentioned above, NH_3_ and H_2_S adsorptions lead to a little charge in the band gap of the pristine PG, which demonstrates that PG has poor sensitivity to NH_3_ and H_2_S. However, the band gap of B-, N-, and Si-PG undergo great variation after NH_3_ adsorption, as shown in Figure 6e,f,h, implying that NH_3_ adsorption has a huge impact on the resistivity of B-, N-, and Si-doped PG, thus these doped PG exhibits high NH_3_ sensitivity. Furthermore, the adsorption strength of NH_3_ on the B-, N-, and Si-PG surface is much larger than that of H_2_S and SO_2_ on the same substrates (see Table 3). Those results indicate that B-, N-, and Si-PG show excellent NH_3_ selectivity and sensitivity and could be a promising gas sensor for NH_3_ detection. In addition, compared with the Al-PG, the B-PG also has good H_2_S sensitivity due to the obvious change of band gap, as displayed in Figure 6i. Nonetheless, the band gap of Al-PG and B-PG remains unchanged in spite of SO_2_ chemisorption (Figure 6k,l), which suggests that Al- and B-PG could not be the SO_2_ sensing materials.

**Figure 7:**
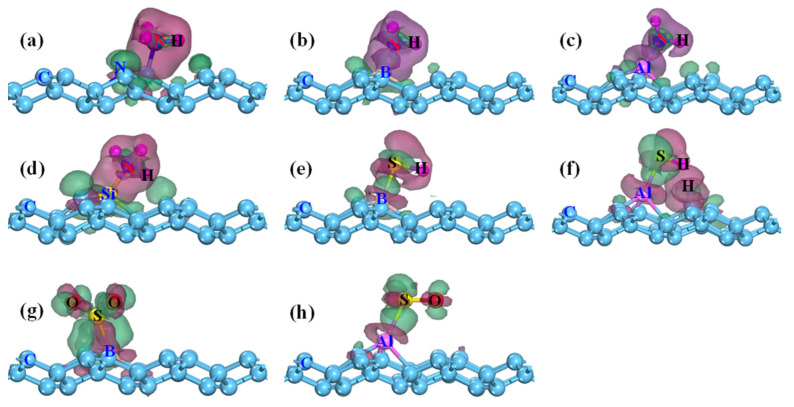
Difference charge density of different adsorption systems (**a**) NH_3_@N-PG, (**b**) NH_3_@B-PG, (**c**) NH_3_@Al-PG, (**d**) NH_3_@Si-PG, (**e**) H_2_S@B-PG, (**f**) H_2_S@Al-PG, (**g**) SO_2_@B-PG, and (**h**) SO_2_@Al-PG. The green (red) region is electron accumulation (depletion), and the isosurface is ±0.02 e/Å^−3^.

Figure 7 displays the difference charge density of NH_3_, H_2_S, and SO_2_ adsorbed the doped PG. For the NH_3_ adsorption, one sees that the charge accumulation primarily appears around the N, B, Al, and Si atoms, while the charge depletion occurs in the vicinity of the NH_3_ molecule (Figure 7a–d). The Hirshfeld charge analysis indicates the NH_3_ acts as an electron donor with a large charge transfer from NH_3_ to N-, B-, Al-, and Si-doped PG of 0.465 e^−^, 0.406 e^−^, 0.312 e^−^, and 0.354 e^−^, respectively. Those results also demonstrate that NH_3_ is chemically adsorbed on N-, B-, Al-, and Si-doped PG. The CDD plot of H_2_S@B-PG shows similar results as displayed in Figure 7e. However, for the SO_2_@B-PG and SO_2_@Al-PG systems, the charges are mainly accumulated around the SO_2_ molecule, while the charges are depleted near the B and Al dopants, thus the SO_2_ acts as an electron acceptor and obtains the charge from B-PG and Al-PG by 0.105 e^-^ and 0.203 e^−^ (see Table 3). Considering the large adsorption strength and charge transfer, the SO_2_ adsorbed on the B- and Al-doped PG also belongs to chemisorption.

**Figure 8:**
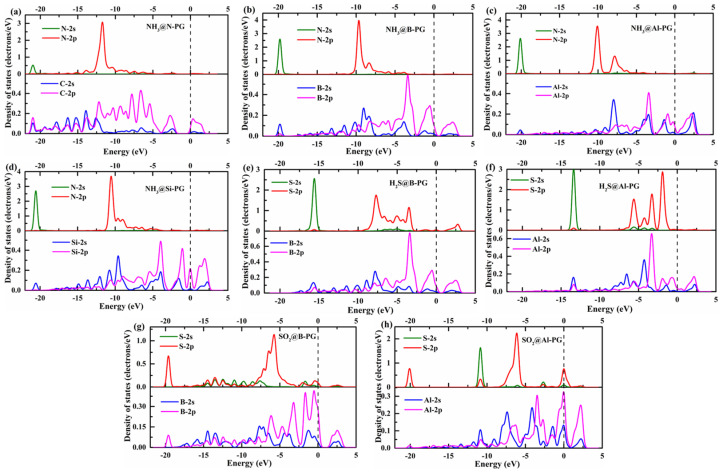
The DOSs of the N (S) atom of NH_3_ (H_2_S/SO_2_) and its nearest neighbor dopant atoms for various adsorption systems, (**a**) NH_3_@N-PG, (**b**) NH_3_@B-PG, (**c**) NH_3_@Al-PG, (**d**) NH_3_@Si-PG, (**e**) H_2_S@B-PG, (**f**) H_2_S@Al-PG, (**g**) SO_2_@B-PG, and (**h**) SO_2_@Al-PG. The dashed line is the Fermi level.

In order to further understand the micro-interactions of the gas-substrate, we calculated the DOSs of the N (S) atom of NH_3_ (H_2_S/SO_2_) and its nearest neighbor dopant atoms for various adsorption systems, as displayed in Figure 8. For the NH_3_@N-PG system, the NH_3_ is adsorbed on top of its nearest neighbor C atom, and the obvious orbital hybridizations between C-2p and N-2p of NH_3_ can be observed in the range of −12.5 eV to −10 eV, while the relatively weak interactions between C-sp and N-2s are found at −21.05 eV as shown in Figure 8a. For the NH_3_@B-PG, NH_3_@Al-PG, and NH_3_@Si-PG systems (Figure 8b–d), there are significant hybridizations between N-2p of NH_3_ and B-, Al-, and Si-sp orbitals ranging from −10.0 eV to −5.0 eV. Moreover, there are also weak hybridizations of N-2s and B-, Al-, and Si-2s around −20.0 eV. In summary, the strong interactions between the N atom of NH_3_ and its nearest neighbor atoms result in NH_3_ chemisorption. As for the H_2_S@B-PG and H_2_S@Al-PG, the strong adsorption strength of the H_2_S molecule is primarily contributed by the orbital interactions between S-2p of H_2_S and B- and Al-sp in the range of −10.0 eV to 0.0 eV (Figure 8e–f). By contrast, the strong orbital hybridizations between S-2p of SO_2_ and B- and Al-sp occur in the larger range of −10.0 eV to 2.5 eV for the SO_2_@B-PG and SO_2_@Al-PG systems, which leads to the SO_2_ molecule being chemically adsorbed over the B- and Al-doped PG.

### 3.4. Recovery of Doped PG after Sensing Toxic Gases

**Figure 9:**
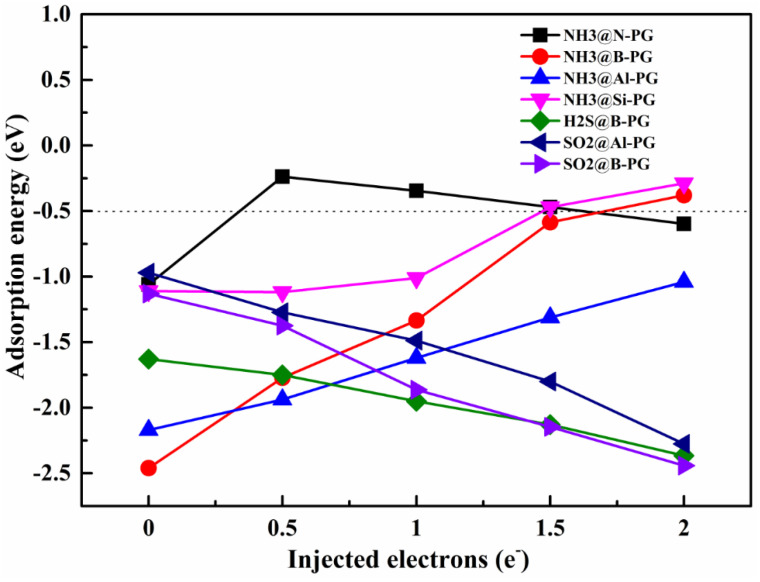
Adsorption energies of NH_3_, H_2_S, and SO_2_ over doped PG as a function of the injected negative charges.

The short recovery time at room temperature is also of great importance for a good sensing material. Consequently, the feasibility of the toxic gases (NH_3_, H_2_S, and SO_2_) desorbed from the N-, B-, Si-, and Al-doped PG was explored in this section. As displayed in Table 3, the adsorption energies of NH_3_, H_2_S, and SO_2_ on the doped PG with chemisorption are all lower than −0.97 eV. As known, the gas molecule can be released from a solid material when the corresponding E_ads_ is higher than −0.50 eV [36]. Accordingly, those toxic gases cannot be desorbed immediately after adsorption, and the recovery time becomes very long at room temperature. Recently, the introduction of a negative charge could effectively modulate the adsorption strength of a gas molecule on the substrate [37,38,39]. In this regard, the adsorption energies of NH_3_, H_2_S, and SO_2_ on the doped PG with a different negative charge were calculated, and the results are shown in Figure 9. With the increase in injected negative charge, the adsorption strength of NH_3_ on B-, Al-, and Si-doped PG is gradually decreased, while that of H_2_S and SO_2_ on B- and Al-doped PG is gradually increased. This indicates that the H_2_S or SO_2_ removal from the B-PG and Al-PG surface becomes more difficult after the introduction of electrons due to the stronger adsorption strength. Nevertheless, the adsorption energies of NH_3_@B-PG and NH_3_@Si-PG are increased from −2.46 eV to −0.38 eV and from −1.11 eV to −0.29 eV after adding 2.0 e^−^ into the systems. For the NH_3_@N-PG system, the adsorption energy is also remarkably changed from −1.06 eV to −0.24 eV (−0.35 eV) after injecting 0.5 e^−^ (1.0 e^−^) into the N-PG substrate. Thus, we can conclude that the chemically adsorbed NH_3_ molecule on the N-, B-, and Si-doped PG could be easily desorbed by controlling the number of injected electrons into the systems. Considering the N-, B-, and Si-doped PG also exhibit good selectivity and sensitivity toward the NH_3_ molecule as discussed above, we can therefore say that the N-, B-, and Si-doped PG could be a promising sensing material for NH_3_ detection.

## 4. Conclusions

In summary, the adsorption behaviors and gas sensing properties of the pristine and N-, B-, P-, Al-, and Si-doped PG toward three toxics gases (NH_3_, H_2_S, and SO_2_) were studied by DFT calculations in this work. In addition, the adsorption characteristics, electronic properties, and the sensing mechanism were also discussed. The results of formation energy show that the substitution of the C1 atom by N, B, P, Al, or Si is energetically favorable. The pristine PG exhibits poor sensing ability of the NH_3_, H_2_S, and SO_2_ molecules due to weak adsorption strength. In contrast, doping B, N, Al, or Si atoms into PG nanosheets can obviously improve the NH_3_ adsorption strength, while only the doping of the B and Al atoms enhance the interactions between the H_2_S and SO_2_ molecules and PG, which was further verified by the analysis of DOSs, CDD, and the charge transfer. Additionally, the adsorption strength of NH_3_ on B-, N-, and Si-doped PG is much larger than that of H_2_S and SO_2_ on the same substrates, demonstrating that the B-, N-, and Si-doped PG have good NH_3_ selectivity from the mixtures of H_2_S and SO_2_. In addition, doped PG exhibits high NH_3_ sensitivity due to its great variation of resistivity after NH_3_ adsorption. More interestingly, the chemically adsorbed NH_3_ molecule on the N-, B-, and Si-doped PG could be effectively desorbed by controlling the number of injected electrons into the systems. Therefore, the N-, B-, and Si-doped PG can be the potential materials to develop NH_3_ sensors with high sensitivity and selectivity.

## Figures and Tables

**Table 1:** The formation energy of penta-graphene with different dopants.

Structures	E_for_/eV	
	sp^2^ Hybridized Carbon Atom (C1 Site)	sp^3^ Hybridized Carbon Atom (C2 Site)
B-doped PG	+0.92 (+0.67) [17]	+1.00 (+0.74) [17]
N-doped PG	−0.10 (−0.01) [17]	+1.80 (+1.90) [17]
P-doped PG	−0.14	+0.95
Si-doped PG	+0.87	+0.96
Al-doped PG	+1.51	+3.23

**Table 2:** Adsorption energy (E_ads_), the Hirshfeld charge transferred from PG to gases (Q_T_), adsorption distance (d), and band gap (E_g_) of different gases adsorbed on pristine PG.

Gas Molecules	E_ads_/eV	Q_T_/e	d/Å	E_g_/eV
NH_3_	−0.382	0.058	2.970 (N-C)	2.35
H_2_S	−0.298	0.040	3.169 (S-C)	2.22
SO_2_	−0.328	−0.072	2.661 (S-C)	1.90

**Table 3:** Adsorption energy (E_ads_) of various adsorption systems, Hirshfeld charge transferred from gases to doped PG (Q_T_), and band gap of the system before adsorption (E_g_) and after adsorption (E_g_’). The positive (negative) Q_T_ means that gases lose (gain) charge.

Structures	NH_3_			H_2_S			SO_2_		
	E_ads_	Q_T_	E_g_ (E_g_’)	E_ads_	Q_T_	E_g_ (E_g_’)	E_ads_	Q_T_	E_g_ (E_g_’)
B-doped PG	−2.46	0.406	1.81/1.23	−1.63	0.360	1.81/1.31	−1.13	−0.105	1.81/1.82
N-doped PG	−1.06	0.465	1.88/2.27	−0.20	0.009	1.88/1.65	−0.42	−0.133	1.88/0.00
P-doped PG	−0.57	0.147	0.00/0.00	−0.40	0.067	0.00/0.00	−0.29	−0.043	0.00/1.33
Al-doped PG	−2.17	0.312	0.54/0.76	−2.87	−0.043	0.54/0.42	−0.97	−0.203	0.54/0.46
Si-doped PG	−1.11	0.354	1.32/0.84	−0.41	0.073	1.32/1.20	−0.35	−0.168	1.32/1.10

## Data Availability

Data available upon request.
